# Influence of Simulated Gastrointestinal Digestion on Phenolic Composition, Bioaccessibility, and Antioxidant Properties of Commercial Wild Rice

**DOI:** 10.3390/molecules31132333

**Published:** 2026-07-03

**Authors:** Asif A. Panchbhaya, Daniela D. Herrera-Balandrano, Beverly Too, Trust Beta

**Affiliations:** Department of Food and Human Nutritional Sciences, University of Manitoba, Winnipeg, MB R3T 2N2, Canada; panchbaa@myumanitoba.ca (A.A.P.); daniela.herrerabalandrano@umanitoba.ca (D.D.H.-B.); toob@myumanitoba.ca (B.T.)

**Keywords:** wild rice (*Zizania palustris* L.), in vitro digestion, phenolic acids, bioaccessibility, antioxidant capacity

## Abstract

Wild rice (*Zizania palustris* L.; WR) is a nutrient-dense whole grain, naturally rich in phenolic acids with established antioxidant and health-promoting properties. This study investigated the effect of cooking and in vitro gastrointestinal digestion on the phenolic composition, bioaccessibility, and antioxidant capacity of commercial WR samples. Free phenolics predominated over bound forms in both raw and cooked samples, indicating that WR is a rich source of extractable phenolics. Cooking reduced total phenolics by 17–23% across WR varieties. Among individual compounds, p-hydroxybenzoic acid (up to 58.3%) and caffeic acid (up to 56.5%) exhibited the greatest bioaccessibility indices, while ferulic acid remained largely insoluble. In vitro gastrointestinal digestion facilitated the release of some bound phenolics, particularly during the intestinal phase, resulting in a total phenolic content (TPC) bioaccessibility of up to 22.61%. However, antioxidant activities, as measured by DPPH and ABTS assays, declined compared to cooked samples (5.53% and 9.34%, respectively). These findings reveal dynamic changes in phenolic composition and bioaccessibility during cooking and digestion, with the intestinal phase being pivotal for releasing certain bound phenolics. This underscores WR’s promise as a functional food source for phenolics, while highlighting the importance of evaluating food bioactives under physiologically relevant conditions.

## 1. Introduction

Wild rice (*Zizania palustris* L.; WR) is a nutrient-rich aquatic cereal grain cultivated in North America and East Asia, recognized for its notable antioxidant activity and health-promoting effects [1]. As a promising functional food, WR is rich in protein, minerals, and vitamins while remaining low in fat. Its diverse phytochemical profile includes phenolic acids, flavonoids, phytosterols, γ-oryzanol, and γ-aminobutyric acid [2]. The phenolic compounds and other phytochemicals in WR have been linked to the prevention of chronic diseases and may offer a range of benefits, including alleviation of insulin resistance and lipotoxicity, prevention of atherosclerosis, and anti-inflammatory, anti-allergic, antihypertensive, and immunomodulatory effects [2,3].

However, the health benefits of phenolic-rich foods depend critically on their bioaccessibility, which refers to the proportion of compounds released from the food matrix during digestion and available for absorption in the gastrointestinal tract [4,5]. Phenolic compounds must be exposed to gastrointestinal conditions to exert their biological properties, but the digestive process can significantly alter both their composition and activity [5]. Because digestion can substantially impact the stability and bioaccessibility of phenolic compounds, in vitro simulated gastrointestinal digestion methods have been developed to evaluate these critical changes [4,6].

Research on pigmented rice and related grain products has shown that the behavior of phenolic compounds during digestion is both compound-specific and matrix-dependent [7,8]. Different phenolic classes exhibit distinct bioaccessibility patterns at each stage of digestion; for example, phenolic acids and lignans often demonstrate higher cellular bioaccessibility than other phenolic types [8]. Notably, antioxidant capacity is frequently reduced between the gastric and intestinal phases, highlighting the difference between chemical stability and biological efficacy [7]. Despite growing evidence on the fate of phenolic compounds during digestion, a comprehensive characterization of how simulated gastrointestinal digestion influences the phenolic composition and antioxidant properties of commercial WR remains limited.

This study investigates the influence of simulated gastrointestinal digestion on the phenolic composition and antioxidant properties of commercial WR, to elucidate which phenolic compounds remain stable and bioaccessible throughout digestion, and which are susceptible to degradation or transformation. The findings will contribute to a more comprehensive understanding of the functional value of WR as a dietary source of bioactive compounds and may inform strategies to optimize the health benefits associated with its consumption.

## 2. Results and Discussion

### 2.1. Impact of Cooking and In Vitro Digestion on Phenolic Content and Composition

**Figure 1:**
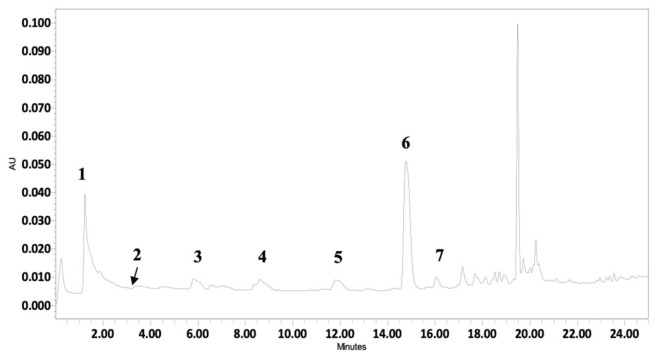
HPLC chromatogram of phenolic acids in commercial WR samples. Identified peaks: 1, gallic acid; 2, p-hydroxybenzoic acid; 3, caffeic acid; 4, vanillic acid; 5, p-coumaric acid; 6, ferulic acid; and 7, sinapic acid. Retention times correspond to phenolic acid standards.

Phenolic acids can be structurally classified as hydroxybenzoic or hydroxycinnamic acids [9]. In this study, three commercial WR varieties (EG: Epigrain, SY: SunYeah, and FL: Floating Leaf) were analyzed and found to contain primarily three hydroxybenzoic acids and four hydroxycinnamic acids (Figure 1). Identification was achieved by comparing the chromatographic peaks with authentic phenolic acid standards corresponding to peaks 1–7, namely gallic, p-hydroxybenzoic, caffeic, vanillic, p-coumaric, ferulic, and sinapic acids. Quantitative analysis was performed by integrating the area under the curve for each peak using an HPLC system equipped with a photodiode array detector. It should be noted that, although several additional peaks were observed in the chromatograms, only seven phenolic acids were identified and quantified based on available reference standards. Thus, other phenolic compounds present in WR may remain unidentified, and further studies using advanced techniques or a broader range of standards are needed to fully characterize the phenolic profile.

#### 2.1.1. Effect of Digestion Phases on Phenolic Acids

The digestive process has a significant effect on phenolic content, as phenolics are hydrolyzed and metabolized by the environment or microbial flora. The amounts, antioxidant properties, and structures of compounds are altered as they pass through the digestive tract as a result of enzymatic activities, pH alterations, and metabolic activities of the intestinal flora [10].

Gallic acid concentrations in the raw free extracts of EG, FL, and SY were 436.60, 435.24, and 162.30 mg/kg, respectively (Table 1). These levels decreased significantly after cooking and throughout digestion, with the oral phase resulting in an approximately tenfold reduction. Further decreases occurred during the gastric phase, followed by slight increases in the intestinal phase for FL and SY. In contrast, EG exhibited slightly higher gallic acid levels in both the intestinal free and bound fractions. Overall, free extracts contained higher gallic acid concentrations than bound extracts, and the levels were generally higher than those reported by Chu et al. [9], possibly due to geographical growing conditions.

Cooking increased free p-hydroxybenzoic acid in EG from 13.45 to 36.19 mg/kg (93% increase), which further increased to 55.47 mg/kg after oral digestion, highlighting the impact of α-amylase. Gastric digestion reduced p-hydroxybenzoic acid levels; however, the intestinal phase resulted in a subsequent increase. In FL, the highest p-hydroxybenzoic acid concentration was observed in the oral phase (75.24 mg/kg), decreasing to 11.49 mg/kg in the gastric phase and rising again to 28.84 mg/kg in the intestinal phase. SY followed a similar trend but at lower concentrations.

Caffeic acid content decreased slightly after cooking in EG and FL but increased during oral digestion. Gastric digestion led to a reduction, followed by a subsequent increase in the intestinal phase. In SY, the highest caffeic acid content was observed in the intestinal-bound extract, followed by the oral-phase extract, with no significant difference between the gastric and intestinal free extracts.

Vanillic acid in EG and FL peaked after oral digestion, decreased in the gastric phase, and increased again in the intestinal phase. In SY, vanillic acid was highest in the intestinal bound extract. p-Coumaric acid in EG and FL increased after oral digestion but decreased during the gastric phase and increased again in the intestinal phase. In SY, the highest p-coumaric acid content was observed in the intestinal bound extract, followed by the oral phase, with lower levels in the gastric phase and a slight increase in the intestinal phase.

Sinapic acid was highest after oral digestion, decreased during the gastric phase, and increased again in the intestinal phase across all varieties. Ferulic acid was significantly higher in the intestinal-bound fraction for all varieties. In FL and SY, ferulic acid decreased in the gastric free fraction and increased in the intestinal phase, whereas in EG, it was higher in the gastric phase but lower in the intestinal phase, with no significant differences overall.

The higher concentrations of p-hydroxybenzoic, gallic, caffeic, sinapic, and p-coumaric acids after the oral phase may be attributed to their rapid release from the WR matrix during salivary hydration. Salivary amylase may have facilitated the hydrolysis of matrix macronutrients, thereby contributing to greater release of phenolics during the oral phase [10]. A previous study reported a 91% bioaccessibility of free phenolics following oral digestion of bread [11]. Similarly, a study on legumes reported higher bioaccessibility of phenolic acids during the oral phase in four bean types compared with the gastric and intestinal phases [12].

The decrease in phenolic acid content following gastric digestion may be attributed to several factors, including the formation of phenolic-protein complexes, which may have reduced freely extractable phenolics during digestion [11]. Secondly, the changes in pH during gastric digestion may have promoted adsorption of phenolic acids to cellulose and xylan [2], limiting their bioaccessibility, especially around pH 2. Adsorption onto dietary fiber may have reduced the measurable concentration of phenolic acids [13]. The acidic gastric environment (pH ~2) not only promotes protein-phenolic binding and fiber adsorption but may also accelerate hydrolytic or oxidative degradation of certain phenolic acids, reducing their measurable concentrations. Additionally, the reduction in caffeic and p-Coumaric acid content during gastric digestion may be explained by their possible transformation into compounds not quantified as original phenolic acids. Liu et al. [14] reported the fate of hydroxycinnamic, caffeic and p-coumaric acids in gastric digestion as chemical transformations, whereby p-coumaric acid underwent gastric decarboxylation and caffeic acid generated several derivative compounds through oxidation, dehydration, and esterification reactions.

The slight increase in p-hydroxybenzoic, gallic, caffeic, sinapic, and p-coumaric acids during intestinal digestion may be attributed to the continued disruption of the WR cell matrix by pancreatic enzymes and bile salts, which facilitates the release of phenolic acids previously bound to cell-wall polysaccharides and protein complexes. The hydrolysis of ester and glycosidic linkages, together with increased solubilization at neutral to alkaline pH, may have contributed to extractability efficiency. Similar increases in phenolic bioaccessibility during intestinal digestion have been reported in wheat, where hydroxycinnamic acids were progressively liberated from the grain matrix, resulting in increased bioaccessible concentrations at the end of the intestinal phase [15]. In the intestinal phase, the shift to neutral or slightly alkaline pH, together with the action of pancreatic enzymes and bile, enhances both the solubility and hydrolysis of phenolic conjugates, further increasing the release of free phenolic acids.

It should be emphasized that, although both free and bound phenolic acids were quantified in this study, only selected phenolic acids were targeted. Thus, observed changes may also reflect conversion into undetected derivatives, conjugates, glycosides, esters, or degradation products. As such, the trends reported likely represent a combination of matrix release, hydrolysis, degradation, altered solubility, and transformation processes, rather than changes in only the originally quantified phenolics.

#### 2.1.2. Comparative Analysis of Phenolic Acids Among WR Varieties

In the raw free extracts, gallic acid levels were significantly higher in EG and FL compared to SY, while no differences were observed among the samples in the raw bound extracts. Cooking decreased free gallic acid content by 19–50%, whereas bound gallic acid levels after cooking did not differ significantly from those in the raw bound extracts. During the oral phase, gallic acid decreased approximately tenfold in FL and SY and 24-fold in EG. The gastric phase further reduced free extract levels by approximately half relative to the oral phase, while the intestinal phase significantly increased free gallic acid. This increase may be attributed to NaOH-mediated hydrolysis of ester and ether linkages, which is known to release bound phenolics, although this mechanism was not assessed in this study.

For p-hydroxybenzoic acid, the free raw extract in FL was significantly higher than in EG and SY. Cooking increased p-hydroxybenzoic acid content in SY by 19%, but levels decreased by 58% after intestinal digestion, reflecting the influence of pH on phenolic stability. After oral digestion, p-hydroxybenzoic acid levels increased significantly, followed by a decrease during the gastric phase and a subsequent increase in the intestinal phase. EG exhibited a twofold increase after cooking, followed by a 19% decrease after digestion. In FL, p-hydroxybenzoic acid decreased by 26% after digestion, with no significant effect of cooking.

Caffeic acid content in EG remained relatively unchanged by cooking and digestion, whereas in SY and FL, digestion reduced caffeic acid levels by approximately threefold. This reduction may be attributed to changes in solubility, bioaccessibility, and chemical structure during gastrointestinal digestion. Caffeic acid is primarily absorbed in the gastric environment, with decreased levels in the intestinal phase, possibly involving monocarboxylic acid transporters [16]. In vitro digestion has been reported to reduce phenolic content and antioxidant activity [17,18].

Vanillic acid was unaffected by cooking but decreased during digestion in FL and SY by approximately fourfold and sixfold, respectively, whereas EG showed no significant reduction. The retention of vanillic acid and other phenolics after gastrointestinal digestion may reduce degradation at high pH values despite their inherent instability [19].

Free p-coumaric acid decreased in all varieties after cooking and digestion; however, a significant increase in the bound fraction was observed after intestinal digestion. The accumulation of insoluble p-coumaric acid in the bound fraction may contribute to its bioaccessibility during colonic microbial fermentation, as reported in wheat studies [20], potentially conferring anti-inflammatory effects.

Ferulic acid was abundant in the bound fraction across all samples and digestion phases, peaking in the intestinal phase, likely due to alkaline hydrolysis. Free ferulic acid was highest before cooking and digestion but decreased approximately twelvefold thereafter. As a hydroxycinnamic acid, ferulic acid is often ester-linked to polysaccharides and released under alkaline conditions [21]. Sinapic acid was highest in the orally digested extracts and remained elevated in the intestinal-bound fraction, possibly due to temperature and pH effects weakening the fiber–protein matrix.

Comparatively, total phenolic content in raw samples was highest in EG (801.10 mg/kg), followed by FL (677.25 mg/kg) and SY (355.02 mg/kg). After digestion, total phenolics in EG and FL decreased by 26.5% and 27.5%, respectively, whereas SY exhibited a 5.5% increase. Processing significantly influenced phenolic acid content, depending on thermal stability and interactions with cell wall components. Free gallic, ferulic, sinapic, and p-coumaric acids decreased after cooking, consistent with reports that thermal processing degrades phenolic compounds [22]. Gastrointestinal digestion may either increase or decrease phenolic content depending on pH stability. The intestinal phase confirmed the presence of hydroxycinnamic acids (ferulic, sinapic, and p-coumaric), which may exert beneficial effects in the colon, including reduced inflammation and improved gut health.

### 2.2. Total Phenolic Content (TPC) in WR Samples

Table 2 illustrates the total phenolic content (TPC) in EG, SY, and FL across different processing states (raw and cooked) and digestion phases (oral, gastric, and intestinal), highlighting both free and bound phenolic fractions. Cooking significantly reduced TPC in the free phenolic fraction for all varieties. For instance, EG decreased from 17.27 mg GAE/g (raw) to 11.95 mg GAE/g (cooked), with similar patterns observed in SY and FL. This trend is consistent with findings in pigmented and wild rice, where cooking typically reduces free phenolic compounds due to leaching, chemical degradation, or transformation [7]. However, a recent study on commercial WR (*Zizania aquatica* L.) reported that TPC remained largely unchanged after boiling, with approximately 74% of flavonoids, proanthocyanidins, and anthocyanins retained [23]. These discrepancies highlight the impact of variety, processing conditions, and intrinsic grain characteristics on phenolic retention.

During simulated gastrointestinal digestion, a significant reduction in free phenolics was observed, particularly after the gastric phase. EG, SY, and FL showed marked decreases to 1.92, 0.94, and 2.56 mg GAE/g, respectively. This reduction reflects the destabilizing effects of acidic pH and enzymatic degradation in the gastric environment, consistent with previous studies on cereals reporting diminished free phenolics during digestion [4].

In contrast, the bound phenolic fraction exhibited a different pattern. Although initial values in raw and cooked samples were lower, substantial increases were observed after the oral and intestinal phases. For example, bound phenolic content in FL increased from 4.01 mg GAE/g (raw) and 4.75 mg GAE/g (cooked) to 11.5 mg GAE/g after the oral phase and 9.38 mg GAE/g after the intestinal phase. Similar trends were observed in EG and SY. This suggests that digestive processes, particularly in the intestinal phase, facilitate the release of phenolic compounds previously bound to the rice matrix, thereby enhancing their extractability and potential bioaccessibility [7,24]. These findings are consistent with studies on other cereals, in which in vitro digestion disrupts cell wall structures and releases bound phenolics, thereby increasing antioxidant potential after digestion [25].

Among the varieties, EG exhibited the highest initial free phenolic content, while FL and SY showed greater increases in bound phenolics following digestion. These differences may reflect variations in phenolic composition, binding interactions within the matrix, or structural characteristics inherent to each variety [26,27].

In summary, TPC in WR is significantly affected by both processing and digestion. Cooking and gastric digestion reduce free phenolic content, whereas intestinal digestion promotes the release of bound forms, potentially enhancing bioaccessibility and biological activity. These findings highlight the importance of considering both free and bound fractions, as well as processing and digestion effects, when evaluating the nutritional quality and health-promoting potential of WR.

### 2.3. Antioxidant Activities of WR Samples Before and After In Vitro Digestion

Table 2 presents the antioxidant activities of WR samples as measured by the DPPH and ABTS assays. For both assays, the highest radical scavenging activities were observed in the free phenolic fractions of raw samples (EG Raw Free: 91.60 and 83.80 µmol TE/g DW for DPPH and ABTS, respectively). Cooking resulted in a significant reduction in antioxidant capacity in both free and bound phenolic fractions. The bound fractions consistently exhibited lower antioxidant activities than the corresponding free fractions in both raw and cooked states. Following in vitro digestion, a further decline in antioxidant activity was observed for both DPPH and ABTS assays. Within the intestinal stage, the bound fractions generally exhibited lower antioxidant values compared to the corresponding free fractions. However, for EG, the intestinal-bound ABTS value (11.80 µmol TE/g DW) was slightly higher than the intestinal-free fraction (10.80 µmol TE/g DW), as shown in Table 2.

These results indicate a strong association between free phenolic content and antioxidant capacity, as free fractions consistently exhibited the highest radical scavenging activities. This finding aligns with previous studies demonstrating that free phenolics are more bioaccessible and play a greater role in antioxidant properties [28]. Cooking may both release phenolics by breaking down cellular structures and degrade thermally sensitive compounds; however, the overall effect observed here was a reduction in total antioxidant capacity, particularly in the free fraction [29].

Collectively, the consistent trends across both DPPH and ABTS assays highlight the substantial impact of processing and gastrointestinal digestion on the antioxidant potential and functional properties of WR.

### 2.4. Bioaccessibility and Recovery Indexes of WR

Table 3 presents the bioaccessibility, recovery, and insolubility indices of different commercial WR samples. Bioaccessibility values varied considerably among the examined phenolic compounds and WR samples. In EG, p-hydroxybenzoic acid, caffeic acid, and vanillic acid exhibited the highest bioaccessibility (approximately 57–58%), whereas most other compounds, including ferulic and gallic acids, remained below 20%. A similar trend was observed in FL, where p-hydroxybenzoic acid reached 58.3%, while gallic acid remained low (8.6%). In SY, overall bioaccessibility was lower, with vanillic acid (28.8%) and p-coumaric acid (21.1%) showing the highest values.

The high bioaccessibility of p-hydroxybenzoic and vanillic acids, particularly in EG and FL, aligns with previous studies indicating that simpler phenolic acids are more readily released from the rice matrix during gastrointestinal digestion [30]. In contrast, the consistently low bioaccessibility and high insolubility of ferulic acid reflect its predominant presence in the bound form within cereal matrices and its poor release under standard digestive conditions [31].

The insolubility index was highest for ferulic acid (above 97% across all samples), indicating that most of this compound remained bound within the matrix after digestion. Other phenolics, such as caffeic acid in EG (29.6%) and gallic acid in FL (51.2%), exhibited lower insolubility indices, suggesting greater release during digestion. The high insolubility of certain phenolic acids can be attributed to their strong covalent linkage to cell wall components, particularly arabinoxylans and lignin, which limit their bioaccessibility during gastrointestinal digestion [4,31].

Total recovery indices revealed that ferulic acid exceeded 100% in all samples (EG: 616%; SY: 419%; FL: 398%), suggesting extensive release and transformation of phenolic compounds originally present in bound, non-extractable forms within the rice matrix [29,32]. For other phenolics, total recovery ranged from 17% to 190%, with the highest recoveries generally observed for vanillic and gallic acids in FL. These findings highlight limitations of conventional extraction methods in estimating the full nutritional potential of cereal-bound phenolics.

Additionally, TPC and antioxidant assays (DPPH and ABTS) showed low bioaccessibility (generally <23%) but high insolubility indices (mostly >70%), indicating that a substantial proportion of antioxidant potential remains unavailable after digestion. Total recovery indices for these assays were generally below 90% across all samples. The consistently low bioaccessibility observed for antioxidant capacity suggests that a significant portion of WR’s antioxidant potential remains unexploited following digestion. This observation is consistent with previous studies on pigmented WR, where TPC and radical scavenging activities were largely associated with insoluble, non-bioaccessible phenolic fractions [33]. It is important to note that, despite some increase in phenolic release during intestinal digestion, the overall bioaccessibility of total phenolics (14–23%) and antioxidant activity (DPPH < 6%, ABTS < 10%) remained relatively low. This suggests that most phenolic compounds and their antioxidant potential in WR are not bioaccessible under the experimental conditions used in this study.

Collectively, these results confirm that although WR contains a diverse range of phenolic compounds, only a fraction becomes bioaccessible during digestion, with substantial variability among individual phenolics and rice varieties. These findings support ongoing efforts to optimize processing strategies that enhance the release of bound phenolics and improve the nutritional and functional value of whole grain rice products.

While the recovery index provides insight into phenolic release during digestion, several methodological limitations must be considered, especially when values exceed 100%. In this study, simulated gastrointestinal digestion followed by alkaline hydrolysis likely liberated phenolics that were not extractable from the cooked WR baseline [25,34]. Strong alkaline hydrolysis, in particular, can release compounds that would not be liberated under physiological conditions, thereby artificially inflating recovery indices. Similar effects have been observed in other cereal grains and attributed to the breakdown of ester and ether bonds under alkaline conditions [34,35]. Additionally, matrix transformations during digestion, such as cell wall disruption, may alter phenolic extractability, while minor analytical variability could also influence these results [36]. Although established extraction and quantification methods were used, the absence of comprehensive mass balance calculations for all phenolic forms and transformation products is a limitation of this study. These factors should be considered when interpreting and comparing recovery index values across studies, especially for compounds predominantly present in the bound fraction.

### 2.5. Principal Component Analysis (PCA) and Pearson Correlation Analysis of Phenolic Compounds and Antioxidant Activities

**Figure 2:**
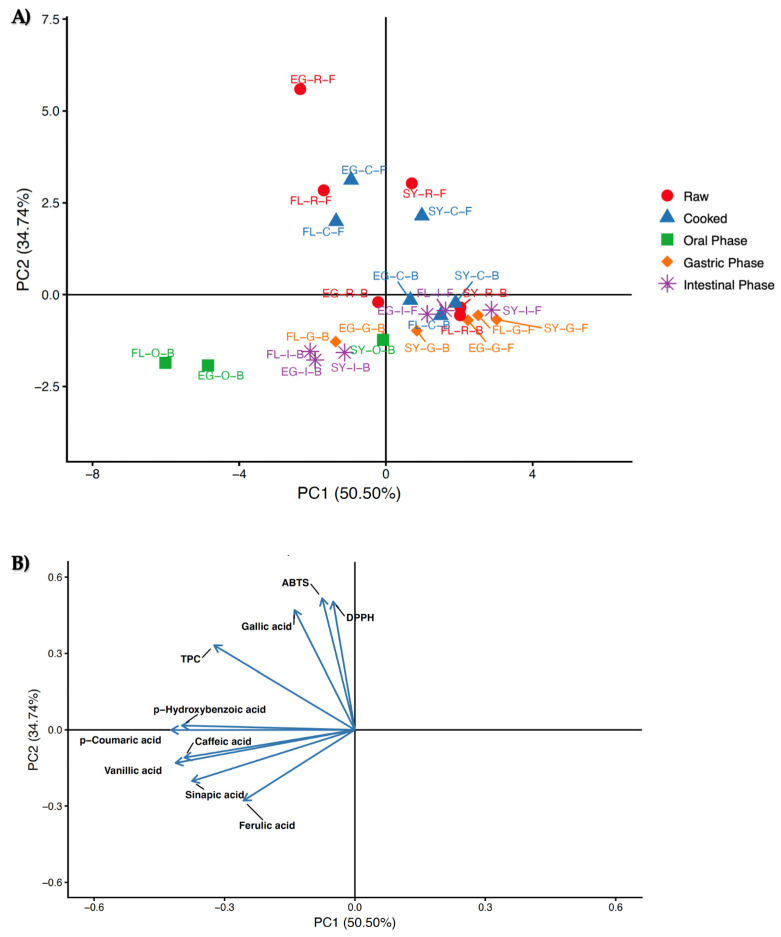
Principal component analysis (PCA) of phenolic composition and antioxidant activities in commercial wild rice samples. (**A**) PCA score plot showing the distribution of samples in principal component space according to processing state and digestion phase. (**B**) PCA loading plot indicating the contribution of each quantified phenolic and antioxidant variable to PC1 and PC2. EG, Epigrain; FL, Floating Leaf Organic; SY, Sun Yeah; O, oral phase; G, gastric phase; I, intestinal phase; R, raw; C, cooked; F, free; B, bound; TPC, total phenolic content; DPPH, 2,2-diphenyl-1-picrylhydrazyl; and ABTS, 2,2′-azino-bis(3-ethylbenzothiazoline-6-sulphonic acid).

Principal component analysis (PCA) (Figure 2) and Pearson correlation analysis (Table 4) were used to evaluate the relationships among individual phenolic compounds, TPC, antioxidant activities, and the effects of processing and digestion phases in WR samples.

The PC scores (Figure 2A) display the distribution of samples in principal components space, while the loading plot (Figure 2B) illustrates the contribution of each phenolic and antioxidant variable to PC1 and PC2. Sample scores, loading values, and variable contribution values are provided in Supplementary Tables S1–S3. The first two principal components, PC1 and PC2, explained 50.50% and 34.74% of the total variance, respectively, accounting for a cumulative 85.24% of the dataset variability. This indicates that most of the variation among samples is captured along two principal axes, facilitating the visualization of complex data patterns. Statistical validation using PERMANOVA confirmed significant group separation among processing and digestion phases (pseudo-F = 4.06, *p* = 0.003; Table S4 and Figure S1).

Raw and cooked samples grouped along the negative axis of PC1 and the positive axis of PC2, and were closely associated with higher TPC, gallic acid, ABTS, and DPPH values, indicating similar phenolic profiles prior to digestion. Gastric- and intestinal-phase samples clustered along the negative axes of both PC1 and PC2 and were linked to increased relative levels of ferulic acid, sinapic acid, and caffeic acid. Oral-phase samples occupied an intermediate position between these groups. Overall, while PCA provides descriptive insights into multivariate relationships, inferential conclusions regarding group separation are based on PERMANOVA results. These observed shifts in sample clustering reflect co-variation among measured variables and sample conditions, which may result from changes in pH, solubility, matrix interactions, or analytical conditions rather than direct evidence of dynamic chemical transformations.

Additionally, Pearson correlation analysis provided further insight into the relationships among variables. Prior to analysis, assumptions of linearity and normality were assessed and found to be acceptable (Table S5). To adjust for multiple comparisons, all p-values were corrected using the Benjamini–Hochberg false discovery rate (FDR) procedure (Table S6). The DPPH and ABTS assays were highly correlated (r = 0.98 ***), indicating similar responses to antioxidant compounds. Both assays also showed strong positive correlations with TPC and gallic acid (ABTS–gallic acid: r = 0.84 ***; DPPH–gallic acid: r = 0.75 ***; TPC–gallic acid: r = 0.79 ***). These associations suggest that gallic acid and overall phenolic content are closely related to antioxidant capacity in WR; however, causality cannot be established, and contributors from other, non-quantified compounds cannot be excluded. In contrast, ferulic acid exhibited weak or negative correlations with antioxidant assays (DPPH: r = −0.37; ABTS: r = −0.33), despite its high concentration in intestinal-phase bound fractions. This pattern is likely an artifact of the pooled data structure, where samples enriched in ferulic acid correspond to later digestion phases characterized by substantially decreased overall antioxidant capacity. Thus, the negative correlations reflect a confounding effect of sample composition rather than an intrinsic property of ferulic acid. Other phenolic acids, including sinapic, vanillic, p-coumaric, p-hydroxybenzoic, and caffeic acids, displayed strong intercorrelations (r = 0.74−0.87 ***), forming a closely associated group. These relationships are likely due to co-extraction patterns or similar structural roles within the WR matrix, rather than direct chemical and biological interactions.

Overall, these findings indicate that antioxidant capacity in WR is primarily associated with gallic acid and TPC, as demonstrated by their clustering patterns and strong correlations with antioxidant assays [26,37,38]. The grouping of raw and cooked samples with TPC and gallic acid highlights the dominance of extractable phenolics prior to digestion. In contrast, the intestinal and gastric phases were characterized by increased relative levels of bound phenolic acids, such as ferulic and sinapic acids, consistent with enzymatic and alkaline conditions that facilitate their release [39]. Although ferulic acid showed weaker correlations with in vitro antioxidant activity, its presence in the bound fraction could be physiologically relevant in the colon [40], where microbial activity may further release these compounds and potentially confer health benefits, including antiglycemic effects [41]. Further studies involving colonic fermentation or in vivo models are needed to confirm these physiological implications. Collectively, PCA and Pearson correlation analyses highlight the dynamic transformation of phenolic compounds during digestion, with gallic acid and total phenolics as prominent contributors to antioxidant capacity.

Despite these insights, it is important to recognize several methodological and analytical limitations. Initially, the static in vitro digestion model used cannot fully replicate the complexity of human gastrointestinal physiology, particularly regarding dynamic enzymatic activity, absorption, and inter-individual variability. Secondly, the simulation of the colonic fermentation stage was not conducted, which means that the potential microbial metabolism and release of bound phenolics in the large intestine were not evaluated. Third, cellular uptake or true bioavailability of phenolic compounds was not assessed. Additionally, the analysis was limited to selected phenolic acids and thus may not capture the full spectrum of bioactive phenolics present in WR. Ultimately, PCA and correlation analyses are constrained by the variables included and may not comprehensively represent all relationships within the dataset.

## 3. Materials and Methods

### 3.1. Chemicals and Reagents

Simulated digestive fluids were prepared using analytical-grade salts (Fisher Scientific, Ottawa, ON, Canada). Potassium chloride (KCl), potassium phosphate monobasic (KH_2_PO_4_), sodium bicarbonate (NaHCO_3_), ammonium carbonate ((NH_4_)_2_CO_3_), and calcium chloride (CaCl_2_) were purchased from Mallinckrodt Specialty Chemical Co. (Paris, KY, USA). Magnesium chloride hexahydrate (MgCl_2_·6H_2_O) was obtained from Sigma-Aldrich (St. Louis, MO, USA). Porcine bile extract (B8631) and digestive enzymes—including α-amylase from porcine pancreas (13 U/mg, A3176), pepsin from porcine gastric mucosa (≥400 U/mg protein, P7000), and pancreatin from porcine pancreas (8× USP, P7545)—were also purchased from Sigma-Aldrich. Hydrochloric acid (37%, Acros Organics, Fair Lawn, NJ, USA) and sodium bicarbonate (Fisher Scientific, Ottawa, ON, Canada) were used. Solvents for phenolic extraction and HPLC analysis included HPLC-grade methanol and ethyl acetate (Fisher Scientific, Ottawa, ON, Canada), as well as formic acid (Acros Organics). Folin–Ciocalteu reagent and phenolic acid standards (gallic acid, p-hydroxybenzoic acid, vanillic acid, caffeic acid, p-coumaric acid, ferulic acid, and sinapic acid) were obtained from Sigma-Aldrich.

### 3.2. Plant Materials and Treatments

**Figure 3:**
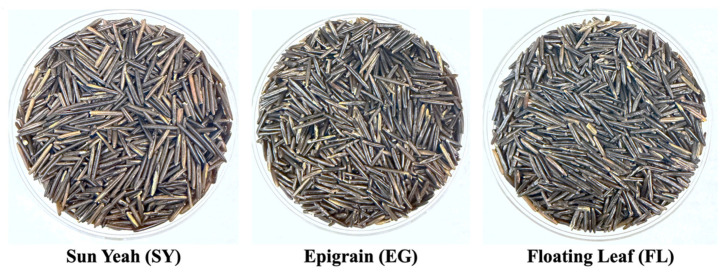
Wild rice (WR) samples: Floating Leaf Organic (FL), Epigrain (EG), and Sun Yeah (SY), all harvested from North America.

Three commercial wild rice brands (*Zizania palustris* L.) were obtained: Floating Leaf Organic (FL) (Northwest Ontario, northern Saskatchewan, and Manitoba), Epigrain (EG) (product of the USA), and Sun Yeah (SY) (northern Saskatchewan) (Figure 3) [42]. Samples were purchased in Manitoba, Canada, in June 2025. WR was cooked in a 1:3 (*w*/*v*) ratio with distilled water in covered beakers. Complete gelatinization was confirmed using the double Petri dish compression test, indicated by the absence of an opaque core. Cooked samples were oven-dried at 40 °C for 3 h, followed by 20 h at 25 °C to achieve constant weight. The dried WR was milled into a fine powder using a coffee grinder (SmartGrind, Black & Decker, Miramar, FL, USA) to ensure uniform particle size for digestion.

### 3.3. In Vitro Digestion

The digestion procedure followed the standardized protocol described by Brodkorb et al. [43], with minor modifications. Simulated salivary fluid (SSF), simulated gastric fluid (SGF), and simulated intestinal fluid (SIF) were prepared as described, except for an adjusted bile extract concentration in the SIF. All solutions and digestive enzymes were freshly prepared before each run, and bile extract (Sigma-Aldrich, B8631) was dissolved in SIF to achieve a final concentration of 0.2% (*w*/*v*) in the overall digestion mixture. Immediately before digestion, the shaking incubator and all digestive solutions were equilibrated to 37 °C. For each run, 2 g of milled WR (*n* = 3) were hydrated with Milli-Q water. A blank digestion (digestive fluids and enzymes without WR) was also prepared under identical conditions.

The hydrated sample was mixed with 2.1 mL of SSF, followed by α-amylase (1500 U/mL in SSF), 0.3 M CaCl_2_, and Milli-Q water to a final volume of 6 mL. The mixture was then vortexed and incubated at 37 °C for 2 min. Subsequently, 4.5 mL of SGF was added to the oral bolus, along with pepsin solution (final enzyme activity: 2000 U/mL), CaCl_2_, Milli-Q water, and 1 M HCl to adjust the pH to 3.0. The mixture was vortexed and incubated at 37 °C for 2 h under constant agitation.

For the intestinal phase, 6.6 mL of SIF was added to the 12 mL gastric bolus, followed by pancreatin (final enzyme activity: 100 U/mL), 1.5 mL of bile solution (0.38 g/mL in SIF), CaCl_2_, 1 M NaHCO_3_ to adjust the pH to 7.0, and Milli-Q water. The mixture was vortexed and incubated at 37 °C for 2 h.

To assess the progression of digestion, triplicate independent samples were collected at the end of each phase (oral, gastric, and intestinal). Digestion was terminated by cooling samples on ice for 10 min, followed by storage at −20 °C until analysis. For post-digestion analysis, frozen digesta samples were thawed at 4 °C, then centrifuged at 12,000 rpm for 15 min at 20 °C. The supernatant (free phenolics) and the pellet (bound phenolics) were collected separately for extraction. For the oral phase, the small hydration volume resulted in insufficient supernatant for reliable analysis of the free phenolic fraction; however, the pellet was subjected to alkaline hydrolysis to quantify bound phenolics, following established protocols.

### 3.4. Extraction of Free and Bound Phenolics

Free phenolics were extracted from the supernatant using three consecutive liquid–liquid extractions with 40 mL of ethyl acetate [44]. Bound phenolic compounds were extracted from the pellets by alkaline hydrolysis: pellets were hydrolyzed with 20 mL of 4 M NaOH, vortexed, and incubated overnight at 4 °C. The resulting hydrolysates were acidified to pH 1.5 using 6 M HCl and extracted three times with ethyl acetate [45]. The upper layers containing free and bound phenolics were collected, combined, and evaporated to dryness using a rotary evaporator. The dried extracts were reconstituted in 80% HPLC-grade methanol, filtered through a 0.22 µm membrane, and stored at −20 °C until HPLC analysis.

### 3.5. HPLC Analysis

Phenolic acids in raw, cooked, and digested samples were analyzed using a Waters 2695 HPLC system (Waters Corp., Milford, MA, USA) equipped with a Waters 996 photodiode array detector and a 100 × 3 mm reversed-phase C18 analytical column (Thermo Scientific, Accucore™, Vilnius, Lithuania) [45]. The column temperature was set at 35 °C, the sample temperature at 15 °C, the flow rate at 0.4 mL/min, and the injection volume at 10 µL. Mobile phase A consisted of 0.1% formic acid in Milli-Q water; mobile phase B was methanol with 0.1% formic acid. The separation was performed using a 25-min linear gradient elution program as follows: 0–3.81 min, 9–14% B; 3.81–4.85 min, 15% B; 4.85–5.89 min, 15% B; 5.89–8.32 min, 15–17% B; 8.32–9.71 min, 17–19% B; 9.71–10.40 min, 19% B; 10.40–12.48 min, 19–26% B; 12.48–13.17 min, 26–28% B; 13.17–14.21 min, 28–35% B; 14.21–15.95 min, 35–40% B; 15.95–16.64 min, 40–48% B; 16.64–18.37 min, 48–53% B; 18.37–22.53 min, 53–70% B; 22.53–25.00 min, 9% B. Detection was performed at 280 and 320 nm. Quantification was achieved by comparison with standard curves (1.563–25 µg/mL), and results were expressed as mg/kg of dry weight (DW).

### 3.6. Total Phenolic Content (TPC)

Total phenolic content (TPC) of free and bound extracts of WR was determined using the Folin–Ciocalteu method as described by Apea-Bah et al. [46]. Briefly, 18.2 µL of extract or standard, 36.4 µL of 10% (*v*/*v*) aqueous Folin–Ciocalteu reagent, and 145.4 µL of 700 mM sodium carbonate were added to a 96-well microplate and incubated in the dark for 1 h. Absorbance was measured at 750 nm using a BioTek ELX800NB microplate reader (BioTek, Vernon Hills, IL, USA). A gallic acid standard curve (0.025–0.150 mg/mL) was used, and the results were expressed as milligrams of gallic acid equivalents per gram of dry weight (mg GAE/g DW).

### 3.7. Antioxidant Activities

#### 3.7.1. DPPH Radical Scavenging Assay

DPPH radical scavenging activity of raw, cooked, and digested WR samples was determined following Apea-Bah et al. [46]. A 60 μM DPPH solution was prepared in methanol and adjusted to an absorbance of 0.700 at 515 nm. In a 96-well microplate, 10 μL of sample or standard was mixed with 190 μL of DPPH solution. The mixture was incubated in the dark at room temperature for 30 min, after which the absorbance was measured at 515 nm using a BioTek ELX800NB microplate reader (Vernon Hills, IL, USA). A Trolox standard curve (0–800 μmol/L) was prepared, and DPPH radical scavenging activity was expressed as micromoles of Trolox equivalents per gram of sample on a dry weight basis (µmol TE/g DW).

#### 3.7.2. ABTS

The Trolox equivalent antioxidant capacity (TEAC) assay was performed according to Apea-Bah et al. [46]. The ABTS radical cation was generated by mixing 16 mM ABTS with 6 mM potassium persulfate and allowing the solution to stand in the dark for 12–16 h. This solution was then diluted with 200 mM phosphate-buffered saline (PBS) to obtain an absorbance of 0.700 at 750 nm. In a 96-well microplate, 10 µL of sample or Trolox standard (100–800 µmol/L) was mixed with 190 µL of ABTS working solution. The mixtures were incubated in the dark at room temperature for 60 min, after which the absorbance was measured at 750 nm using a BioTek ELX800NB microplate reader (Vernon Hills, IL, USA). Antioxidant capacity was expressed as micromoles of Trolox equivalents per gram of sample on a dry weight basis (µmol TE/g DW).

### 3.8. Determination of Bioaccessibility, Insolubility and Recovery Indices

To assess the effects of the in vitro digestion process on commercial WR, bioaccessibility, insolubility, and recovery indices [29,47] were determined for individual phenolic acids, TPC, and antioxidant activities (DPPH and ABTS).

#### 3.8.1. Bioaccessibility Index (%)

The bioaccessibility index represents the proportion of phenolic compounds released into the digestive fluid and potentially available for absorption:

Bioaccessibility Index (%) = (C_soluble_/C_initial_) × 100 (1)

where C_soluble_ is the concentration of the free fraction in the intestinal phase, and C_initial_ is the total concentration (free + bound) in the cooked sample.

#### 3.8.2. Insolubility Index (%)

The insolubility index measures the proportion of bound phenolic compounds remaining after digestion, relative to the total phenolics present in the intestinal phase:

Insolubility Index (%) = (C_insoluble_/(C_soluble_+ C_insoluble_)) × 100 (2)

where C_insoluble_ is the concentration of the bound fraction in the intestinal phase, and C_soluble_ is the concentration of the free fraction in the intestinal phase.

#### 3.8.3. Recovery Index (%)

The recovery index reflects the stability of the compounds throughout the digestive process:

Recovery Index (%) = ((C_soluble_+ C_insoluble_)/C_initial_) × 100 (3)

where C_soluble_ is the concentration of the free fraction in the intestinal phase, C_insoluble_ is the concentration of the bound fraction in the intestinal phase, and C_initial_ is the total concentration (free + bound) in the cooked sample.

### 3.9. Statistical Analysis

All experiments were performed in triplicate, and data are presented as means ± standard deviation (SD). Group comparisons were analyzed using one-way ANOVA followed by Tukey’s test (*p* < 0.05).

Principal component analysis (PCA) variables included TPC, DPPH, ABTS, and individual phenolic acids. Data were mean-centered and scaled to unit variance prior to analysis. The first two principal components accounted for 85.24% of the total variance (PC1 = 50.50%; PC2 = 34.74%). PCA was conducted in R (version 4.4.2; R Foundation for Statistical Computing, Vienna, Austria) using the prcomp function, and plots were generated with the ggplot2 (v4.0.3) and ggrepel (v0.9.8) packages. Statistical validation of group separation was performed using PERMANOVA with 999 permutations on the PC1 and PC2 scores, implemented via the adonis2 function in the vegan package (v2.7-3), yielding a pseudo-F of 4.06 and *p* = 0.003.

Relationships among individual phenolic acids, TPC, DPPH, and ABTS were assessed using Pearson’s correlation coefficient. Statistical significance was determined using the Benjamini–Hochberg false discovery rate (FDR) procedure to correct for multiple comparisons. Correlations with FDR-adjusted *p*-values (q-values) below 0.05 were considered significant; *, **, and *** indicate significance at q < 0.05, 0.01, and 0.001, respectively. Correlation analyses were performed in GraphPad Prism 10.3.1 (GraphPad Software, San Diego, CA, USA).

## 4. Conclusions

This study demonstrates that cooking and in vitro gastrointestinal digestion significantly modulate the phenolic composition and antioxidant capacity of commercial WR. Notably, cooking increased the proportion of phenolic compounds, particularly ferulic acid, in the bound fraction, possibly reflecting associations with starch retrogradation and matrix changes; however, direct measurements of resistant starch were not performed, and this mechanism remains speculative. This phenomenon may help explain the observed limitations in initial bioaccessibility. Although simulated gastrointestinal digestion facilitated the release of some bound phenolics during the intestinal phase, it did not result in increased TPC or antioxidant activity relative to cooked WR.

Multivariate analysis indicated that antioxidant capacity was primarily associated with gallic acid and TPC. While bound ferulic acid was present in significant quantities post-digestion, its potential physiological effects in the lower gastrointestinal tract cannot be inferred from this in vitro model since absorption, metabolism, and in vivo effects were not assessed. HPLC analysis further confirmed that gallic acid was the principal contributor to antioxidant activity following digestion.

Overall, these findings indicate that wild rice is a source of bioactive phenolic compounds and that both processing and in vitro gastrointestinal digestion influence their bioaccessibility. While this study provides insight into compositional changes and potential phenolic release during digestion, the physiological implications of these results cannot be determined from the present in vitro model. These results underscore the importance of considering both processing conditions and gastrointestinal digestion when evaluating the phenolic composition of whole grains. Further research, including studies on food structure, matrix interactions, and dynamic or in vivo digestion systems, is necessary to elucidate the mechanisms of phenolic release and their potential impact on human health.

## Figures and Tables

**Table 1:** Phenolic acid composition of different commercial wild rice during cooking and in vitro gastrointestinal digestion.

WR Sample	State/Phase	Fraction	Gallic Acid	p-Hydroxybenzoic Acid	Caffeic Acid	Vanillic Acid	p-Coumaric Acid	Ferulic Acid	Sinapic Acid	Total Free/Bound	Total Phenolics
			mg/kg								
EG	Raw	F	436.60 ± 63.24 ^a^	18.76 ± 9.31 ^de^	6.77 ± 0.81 ^bc^	12.04 ± 1.42 ^bc^	12.93 ± 1.29 ^b^	12.61 ± 1.64 ^de^	75.63 ± 5.87 ^bcde^	602.34	801.10
		B	31.68 ± 13.94 ^c^	42.22 ± 4.66 ^b^	7.93 ± 3.60 ^b^	14.06 ± 6.30 ^b^	7.98 ± 3.32 ^bcd^	47.48 ± 29.87 ^cd^	47.39 ± 6.77 ^bcde^	198.84	
	Cooked	F	328.17 ± 6.79 ^b^	36.19 ± 0.79 ^bc^	5.68 ± 0.59 ^bc^	10.13 ± 1.03 ^bc^	9.58 ± 0.55 ^bcd^	7.86 ± 0.31 ^e^	66.12 ± 0.49 ^bcde^	463.73	613.26
		B	24.02 ± 2.95 ^c^	12.53 ± 1.76 ^e^	6.55 ± 0.38 ^bc^	11.66 ± 0.67 ^bc^	5.46 ± 0.09 ^def^	35.18 ± 0.46 ^cde^	54.13 ± 7.16 ^cde^	149.53	
	Oral	B	22.85 ± 2.94 ^c^	55.47 ± 6.22 ^a^	15.96 ± 1.23 ^a^	28.02 ± 1.97 ^a^	19.08 ± 2.29 ^a^	119.92 ± 14.32 ^b^	343.21 ± 38.09 ^a^	604.51	604.51
	Gastric	F	9.99 ± 2.23 ^c^	14.60 ± 1.88 ^e^	2.84 ± 0.27 ^c^	5.07 ± 0.47 ^c^	2.46 ± 0.23 ^ef^	2.07 ± 0.24 ^e^	20.60 ± 1.06 ^e^	57.63	300.43
		B	19.41 ± 12.5 ^c^	21.08 ± 6.64 ^de^	6.25 ± 1.45 ^bc^	11.02 ± 2.53 ^bc^	7.98 ± 2.98 ^cd^	50.05 ± 20.34 ^c^	127.01± 7.89 ^bcd^	242.8	
	Intestinal	F	29.57 ± 8.55 ^c^	29.10 ± 1.35 ^cd^	6.66 ± 0.32 ^bc^	11.74 ± 0.55 ^bc^	1.18 ± 0.04 ^f^	1.31 ± 0.04 ^e^	23.22 ± 0.42 ^de^	102.78	588.08
		B	25.75 ± 3.30 ^c^	25.11 ± 2.78 ^cd^	3.90 ± 1.84 ^bc^	25.74 ± 4.47 ^a^	11.27 ± 0.35 ^bc^	263.92 ± 4.17 ^a^	129.61 ± 4.84 ^b^	485.3	
SY	Raw	F	162.30 ± 9.79 ^a^	5.52 ± 0.48 ^cd^	3.52 ± 0.36 ^bcd^	6.35 ± 0.62 ^bcde^	2.90 ± 0.20 ^b^	5.87 ± 0.31 ^de^	68.68 ± 3.17 ^abcd^	255.14	355.02
		B	13.70 ± 3.44 ^cd^	3.90 ± 0.31 ^cd^	3.78 ± 1.80 ^bcd^	6.80 ± 3.14 ^bcd^	2.71 ± 0.87 ^b^	23.94 ± 15.46 ^de^	45.05 ± 8.50 ^cd^	99.88	
	Cooked	F	119.63 ± 0.60 ^b^	6.60 ± 0.93 ^cd^	4.31 ± 0.12 ^bc^	7.74 ± 0.21 ^bc^	2.97 ± 0.06 ^ab^	5.27 ± 0.68 ^de^	51.09 ± 0.38 ^bcd^	197.61	312.35
		B	16.07 ± 5.08 ^cd^	18.33 ± 4.7 ^a^	6.21 ± 1.48 ^b^	10.96 ± 2.59 ^b^	3.92 ± 1.95 ^ab^	84.99 ± 12.99 ^b^	150.87 ± 70.20 ^a^	114.74	
	Oral	B	6.31 ± 1.42 ^d^	1.99 ± 0.99 ^cd^	1.01 ± 0.03 ^d^	1.85 ± 0.05 ^e^	1.11 ± 0.36 ^b^	0.93 ± 0.04 ^e^	17.12 ± 0.04 ^d^	291.35	291.35
	Gastric	F	14.07 ± 3.63 ^cd^	3.52 ± 0.54 ^cd^	3.52 ± 1.57 ^bc^	7.26 ± 2.76 ^bc^	2.92 ± 0.7 ^b^	27.01 ± 17.73 ^de^	55.93± 22.70 ^bcd^	30.32	244.06
		B	12.37 ± 3.23 ^cd^	12.92 ± 1.22 ^b^	5.44 ± 0.77 ^bc^	9.62 ± 1.35 ^bc^	2.45 ± 2.1 ^b^	64.51 ± 7.07 ^cd^	106.43 ± 25.38 ^abc^	213.74	
	Intestinal	F	10.35 ± 1.79 ^cd^	2.32 ± 0.35 ^cd^	1.05 ± 0.29 ^d^	1.94 ± 0.51 ^de^	1.27 ± 0.17 ^b^	1.26 ± 0.43 ^e^	18.45 ± 0.03 ^cd^	36.64	374.43
		B	18.76 ± 1.78 ^c^	15.87 ± 1.26 ^ab^	10.99 ± 0.76 ^a^	19.33 ± 1.33 ^a^	6.23 ± 1.10 ^a^	137.54 ± 21.71 ^a^	129.07 ± 34.68 ^ab^	337.79	
FL	Raw	F	435.24 ± 41.47 ^a^	38.51 ± 1.50 ^b^	9.61 ± 4.00 ^bc^	17.01 ± 7.01 ^bc^	8.89 ± 0.01 ^bc^	9.50 ± 0.09 ^c^	57.26 ± 0.29 ^d^	576.02	677.25
		B	22.04 ± 4.95 ^c^	6.66 ± 5.05 ^e^	4.60 ± 1.22 ^de^	6.43 ± 1.96 ^de^	3.16 ± 1.10 ^def^	20.02 ± 10.85 ^c^	39.36 ± 6.37 ^d^	101.23	
	Cooked	F	350.47 ± 6.71 ^b^	38.01 ± 1.27 ^b^	9.08 ± 2.04 ^bcd^	16.08 ± 3.57 ^bcd^	8.18 ± 0.55 ^bc^	7.03 ± 0.13 ^c^	50.50 ± 0.21 ^d^	479.35	602.08
		B	22.09 ± 2.75 ^c^	11.50 ± 3.28 ^de^	4.60 ± 0.93 ^cde^	8.25 ± 1.63 ^cde^	4.10 ± 0.91 ^def^	27.83 ± 7.11 ^c^	45.08 ± 7.40 ^d^	123.45	
	Oral	B	46.93 ± 11.92 ^c^	75.24 ± 7.97 ^a^	16.47 ± 3.13 ^a^	28.91 ± 5.47 ^a^	19.37 ± 1.43 ^a^	145.44 ± 10.76 ^a^	423.82 ± 39.17 ^a^	756.18	756.18
	Gastric	F	28.41 ± 6.81 ^c^	14.60 ± 1.01 ^de^	1.40 ± 0.17 ^e^	2.54 ± 0.29 ^e^	1.36 ± 0.56 ^f^	1.93 ± 0.15 ^c^	17.89 ± 0.53 ^d^	68.13	453.51
		B	28.10 ± 15.14 ^c^	32.94 ± 6.10 ^b^	8.57 ± 0.91 ^bcd^	15.09 ± 1.59 ^bcd^	10.16 ± 2.51 ^b^	77.65 ± 20.1 ^b^	212.87 ± 40.84 ^b^	385.38	
	Intestinal	F	32.07 ± 2.14 ^c^	28.84 ± 1.69 ^bc^	2.75 ± 1.27 ^e^	4.90 ± 2.23 ^e^	2.68 ± 0.09 ^ef^	3.06 ± 0.43 ^c^	18.18 ± 0.24 ^d^	92.48	490.77
		B	33.23 ± 8.88 ^c^	28.14 ± 0.73 ^bc^	11.81 ± 1.21 ^ab^	20.77 ± 2.11 ^ab^	9.53 ± 0.62 ^b^	131.43 ± 13.49 ^a^	163.38 ± 24.28 ^bc^	398.29	

WR: wild rice; EG: Epigrain, SY: SunYeah, and FL: Floating Leaf, F: free fraction; and B: bound fraction. Values are expressed as mean ± SD (*n* = 3). Lowercase letters indicate significant differences between state and in vitro digestion phases within each WR sample.

**Table 2:** Antioxidant activity of commercial wild rice during cooking and in vitro gastrointestinal digestion phases.

WR Sample	State/Phase	Fraction	TPC	DPPH	ABTS
			mg GAE/g	µmol TE/g	
EG	Raw	F	17.27 ± 1.39 ^a^	91.60 ± 4.40 ^a^	83.80 ± 6.96 ^a^
		B	6.47 ± 1.17 ^cde^	16.70 ± 2.51 ^c^	20.10 ± 2.74 ^c^
	Cooked	F	11.95 ± 0.70 ^b^	51.90 ± 5.35 ^b^	49.40 ± 5.80 ^b^
		B	5.42 ± 0.43 ^df^	20.30 ± 1.31 ^c^	17.30 ± 3.21 ^c^
	Oral	B	9.96 ± 0.65 ^bc^	4.48 ± 0.01 ^de^	5.85 ± 0.02 ^d^
	Gastric	F	1.92 ± 0.25 ^f^	5.35 ± 0.14 ^e^	4.97 ± 0.19 ^d^
		B	5.40 ± 1.72 ^df^	1.69 ± 0.22 ^de^	3.64 ± 0.23 ^cd^
	Intestinal	F	2.78 ± 0.20 ^ef^	8.88 ± 0.44 ^de^	10.80 ± 0.44 ^d^
		B	8.48 ± 0.62 ^bd^	2.68 ± 0.25 ^d^	11.80 ± 0.77 ^cd^
SY	Raw	F	13.26 ± 0.89 ^a^	58.90 ± 0.19 ^a^	54.8 ± 2.01 ^b^
		B	3.12 ± 0.77 ^d^	13.70 ± 1.32 ^d^	12.9 ± 1.70 ^cd^
	Cooked	F	9.50 ± 0.18 ^b^	52.90 ± 1.77 ^b^	43.5 ± 1.68 ^c^
		B	3.18 ± 1.01 ^d^	17.80 ± 2.63 ^c^	15.7 ± 1.25 ^cd^
	Oral	B	7.74 ± 0.56 ^bc^	3.08 ± 0.08 ^fg^	4.19 ± 0.01 ^f^
	Gastric	F	0.94 ± 0.01 ^e^	5.62 ± 0.28 ^g^	4.69 ± 0.34 ^f^
		B	6.43 ± 0.55 ^c^	1.19 ± 0.01 ^ef^	8.60 ± 0.85 ^e^
	Intestinal	F	1.76 ± 0.17 ^de^	7.12 ± 0.13 ^g^	10.70 ± 0.63 ^f^
		B	8.62 ± 0.70 ^b^	1.11 ± 0.05 ^e^	3.33 ± 0.35 ^de^
FL	Raw	F	15.08 ± 0.28 ^a^	30.30 ± 1.43 ^a^	39.50 ± 0.17 ^a^
		B	4.01 ± 1.07 ^e^	5.14 ± 0.58 ^c^	7.99 ± 0.18 ^cd^
	Cooked	F	13.07 ± 0.91 ^ab^	20.60 ± 2.32 ^b^	32.20 ± 5.11 ^b^
		B	4.75 ± 0.70 ^e^	5.37 ± 0.84 ^c^	9.39 ± 0.40 ^c^
	Oral	B	11.5 ± 0.64 ^bc^	5.34 ± 0.03 ^c^	6.93 ± 0.04 ^cd^
	Gastric	F	2.56 ± 0.26 ^e^	3.79 ± 0.18 ^e^	3.39 ± 0.20 ^d^
		B	7.44 ± 1.15 ^d^	0.91 ± 0.01 ^cd^	6.37 ± 0.74 ^cd^
	Intestinal	F	4.03 ± 0.07 ^e^	4.22 ± 0.26 ^de^	6.88 ± 0.18 ^d^
		B	9.38 ± 0.90 ^cd^	1.44 ± 0.02 ^cd^	3.89 ± 0.20 ^cd^

WR: wild rice; EG: Epigrain, SY: SunYeah, FL: Floating Leaf, F: free fraction; and B: bound fraction. Values are expressed as mean ± SD (*n* = 3). Lowercase letters indicate significant differences between state and in vitro digestion phases within each WR sample.

**Table 3:** Bioaccessibility, insolubility, and recovery indices (%) of phenolic compounds and antioxidant capacities in commercial wild rice varieties.

WR Sample	Phenolic Compound/Assay	Bioaccessibility Index (%)	Insolubility Index (%)	Total Recovery Index (%)
EG	*Phenolic compound*			
	Gallic acid	9.07 ± 0.24 ^c^	46.51 ± 2.31 ^e^	17.09 ± 0.93 ^f^
	p-hydroxybenzoic acid	57.74 ± 5.51 ^a^	47.43 ± 4.38 ^e^	110.12 ± 3.95 ^d^
	Caffeic acid	56.51 ± 2.73 ^a^	29.67 ± 1.12 ^f^	80.31 ± 5.09 ^e^
	Vanillic acid	57.79 ± 0.57 ^a^	67.65 ± 0.81 ^d^	175.01 ± 4.75 ^b^
	p-coumaric acid	7.92 ± 0.45 ^c^	90.51 ± 0.17 ^b^	83.57 ± 3.24 ^e^
	Ferulic acid	3.04 ± 0.10 ^c^	99.50 ± 0.02 ^a^	616.32 ± 1.51 ^e^
	Sinapic acid	19.43 ± 1.26 ^b^	84.81 ± 0.27 ^c^	128.58 ± 6.49 ^c^
	*Assay*			
	TPC	16.17 ± 1.49 ^a^	75.32 ± 1.92 ^a^	64.93 ± 1.14 ^a^
	DPPH	3.74 ± 0.57 ^c^	76.81 ± 0.85 ^a^	16.82 ± 0.77 ^c^
	ABTS	7.56 ± 1.33 ^b^	70.39 ± 2.05 ^b^	26.10 ± 2.25 ^b^
SY	*Phenolic compound*			
	Gallic acid	8.39 ± 0.65 ^cd^	63.89 ± 2.88 ^d^	22.70 ± 0.57 ^d^
	p-hydroxybenzoic acid	10.52 ± 2.03 ^bd^	87.22 ± 2.13 ^bc^	178.21 ± 21.60 ^c^
	Caffeic acid	14.11 ± 5.16 ^bd^	91.39 ± 2.16 ^b^	159.23 ± 20.35 ^c^
	Vanillic acid	28.87 ± 10.18 ^a^	90.91 ± 2.14 ^b^	312.74± 39.11 ^b^
	p-coumaric acid	21.18 ± 3.11 ^ab^	84.65 ± 1.81 ^c^	137.39 ± 20.10 ^c^
	Ferulic acid	4.64 ± 0.36 ^d^	98.95 ± 0.67 ^a^	419.15 ± 22.54 ^a^
	Sinapic acid	17.51 ± 0.14 ^abc^	87.56 ± 3.01 ^bc^	140.12 ± 32.41 ^c^
	*Assay*			
	TPC	14.29 ± 2.58 ^a^	83.11 ± 0.94 ^a^	87.3 ± 6.64 ^a^
	DPPH	1.57 ± 0.05 ^c^	86.52 ± 0.36 ^a^	11.7 ± 0.38 ^c^
	ABTS	5.35 ± 0.14 ^b^	76.77 ± 2.77 ^b^	23.8 ± 1.31 ^b^
FL	*Phenolic compound*			
	Gallic acid	8.61 ± 0.67 ^c^	51.29 ± 1.48 ^d^	17.65 ± 0.98 ^d^
	p-hydroxybenzoic acid	58.33 ± 2.69 ^a^	49.42 ± 1.32 ^d^	115.12 ± 2.65 ^c^
	Caffeic acid	21.71 ± 1.91 ^b^	79.82 ± 1.64 ^c^	108.23 ± 14.43 ^c^
	Vanillic acid	19.90 ± 1.76 ^b^	81.39 ± 2.04 ^c^	102.11 ± 6.76 ^c^
	p-coumaric acid	21.93 ± 0.45 ^b^	78.50 ± 0.81 ^c^	102.20 ± 3.65 ^c^
	Ferulic acid	8.95 ± 0.75 ^c^	97.89 ± 1.91 ^a^	398.61 ± 30.11 ^a^
	Sinapic acid	18.99 ± 0.49 ^b^	90.17 ± 0.30 ^b^	190.12 ± 10.76 ^b^
	*Assay*			
	TPC	22.61 ± 1.25 ^a^	69.91 ± 2.91 ^a^	78.15 ± 2.75 ^a^
	DPPH	5.53 ± 0.43 ^c^	74.61 ± 3.23 ^a^	22.41 ± 0.76 ^b^
	ABTS	9.34 ± 1.42 ^b^	63.95 ± 3.71 ^a^	27.23 ± 2.57 ^b^

WR: wild rice; EG: Epigrain, SY: SunYeah, and FL: Floating Leaf. Values are expressed as mean ± SD (*n* = 3). Lowercase letters indicate significant differences among phenolic compounds or assays within each WR sample.

**Table 4:** Pearson correlation matrix displaying the relationships among phenolic acids, TPC, and antioxidant activities (DPPH and ABTS) in commercial WR samples.

	DPPH	ABTS	TPC	Ferulic Acid	Gallic Acid	Sinapic Acid	Vanillic Acid	p-Coumaric	p-Hydroxybenzoic Acid	Caffeic Acid
DPPH	1	0.98 ***	0.64 *	−0.37	0.75 ***	−0.21	−0.13	0.12	0.07	−0.09
ABTS		1	0.72 **	−0.33	0.84 ***	−0.21	−0.06	0.16	0.13	−0.07
TPC			1	0.19	0.79 ***	0.38	0.55 *	0.64 *	0.59 *	0.50 *
Ferulic acid				1	−0.28	0.61 *	0.76 ***	0.52 *	0.32	0.44
Gallic acid					1	−0.13	0.10	0.29	0.36	0.07
Sinapic acid						1	0.80 ***	0.80 ***	0.74 ***	0.83 ***
Vanillic acid							1	0.86 ***	0.76 ***	0.84 ***
p-coumaric								1	0.87 ***	0.80 ***
p-Hydroxybenzoic acid									1	0.80 ***
Caffeic acid										1

Correlations were calculated using pooled data from all wild rice samples across all states (raw and cooked) and digestion phases (oral, gastric, and intestinal), including both free and bound fractions, resulting in a total of 27 observations. *, **, and *** indicate significant correlations at FDR-adjusted *p* < 0.05, 0.01, and 0.001, respectively (Benjamini–Hochberg correction).

## Data Availability

The raw data supporting the conclusions of this article will be made available by the authors on request.

## Supplementary Materials

The following supporting information can be downloaded at: https://www.mdpi.com/article/10.3390/molecules31132333/s1, Figure S1. Principal component analysis (PCA) score plot with 95% confidence ellipses for wild rice samples by processing and digestion phase. Table S1. PCA Sample Scores. Table S2. PCA Variable Loadings. Table S3. Contribution of Variables to Principal Components. Table S4. PERMANOVA results for statistical validation of group separation in PCA space. Table S5. Normality Tests for Phenolic compounds, TPC, and Antioxidant Activities. Table S6. Pearson Correlation Coefficients Among Phenolic Compounds, TPC, and Antioxidant Activities.

## References

[1] Li X., Zhang Y., Wang Y., Wang S., Wang Y., Liu Y., Wu C., Qian J., Zhang C. (2026). Exploring the functional food potential of wild rice (*Zizania palustris*): A comparative analysis of morphological, nutritional, bioactive compounds, and antioxidant activity in whole, milled, and bran fractions. Food Chem. X.

[2] Yu X., Chu M., Chu C., Du Y., Shi J., Liu X., Liu Y., Zhang H., Zhang Z., Yan N. (2020). Wild rice (*Zizania* spp.): A review of its nutritional constituents, phytochemicals, antioxidant activities, and health-promoting effects. Food Chem..

[3] Surendiran G., Alsaif M., Kapourchali F.R., Moghadasian M.H. (2014). Nutritional constituents and health benefits of wild rice (*Zizania* spp.). Nutr. Rev..

[4] Nignpense B.E., Francis N., Blanchard C., Santhakumar A.B. (2021). Bioaccessibility and Bioactivity of cereal Polyphenols: A review. Foods.

[5] Wojtunik-Kulesza K., Oniszczuk A., Oniszczuk T., Combrzyński M., Nowakowska D., Matwijczuk A. (2020). Influence of in vitro digestion on composition, bioaccessibility and antioxidant activity of Food Polyphenols—A Non-Systematic Review. Nutrients.

[6] Morales D., Iriondo-DeHond A., Fernández-Tomé S. (2025). Application of the INFOGEST 2.0 standardized method to study the behavior of phenolic compounds throughout gastrointestinal digestion. Food Chem..

[7] Melini V., Acquistucci R. (2017). Health-Promoting compounds in pigmented thai and wild rice. Foods.

[8] Sęczyk Ł., Sugier D., Świeca M., Gawlik-Dziki U. (2020). The effect of in vitro digestion, food matrix, and hydrothermal treatment on the potential bioaccessibility of selected phenolic compounds. Food Chem..

[9] Chu M.J., Liu X.M., Yan N., Wang F.Z., Du Y.M., Zhang Z.F. (2018). Partial purification, identification, and quantitation of antioxidants from wild rice (*Zizania latifolia*). Molecules.

[10] Ketnawa S., Reginio F.C., Thuengtung S., Ogawa Y. (2022). Changes in bioactive compounds and antioxidant activity of plant-based foods by gastrointestinal digestion: A review. Crit. Rev. Food Sci. Nutr..

[11] Alves G., Lobo L.A., Domingues R.M.C.P., Monteiro M., Perrone D. (2021). Bioaccessibility and Gut Metabolism of Free and Melanoidin-Bound Phenolic Compounds from Coffee and Bread. Front. Nutr..

[12] Li M., Bai Q., Zhou J., de Souza T.S.P., Suleria H.A.R. (2022). In Vitro Gastrointestinal Bioaccessibility, Bioactivities and Colonic Fermentation of Phenolic Compounds in Different Vigna Beans. Foods.

[13] Salazar-López N.J., González-Aguilar G.A., Rouzaud-Sández O., Robles-Sánchez M. (2018). Bioaccessibility of hydroxycinnamic acids and antioxidant capacity from sorghum bran thermally processed during simulated in vitro gastrointestinal digestion. J. Food Sci. Technol..

[14] Liu Y., Li H., Liu G., Zhao Y., Luo Y., Xu W., Song S., Han F. (2026). From simulated digestion to α-glucosidase inhibition: The metabolic fate of wine hydroxycinnamic acids. Curr. Res. Food Sci..

[15] Tomé-Sánchez I., Martín-Diana A.B., Peñas E., Frias J., Rico D., Jiménez-Pulido I., Martínez-Villaluenga C. (2021). Bioprocessed Wheat Ingredients: Characterization, Bioaccessibility of Phenolic Compounds, and Bioactivity During in vitro Digestion. Front. Plant Sci..

[16] Konishi Y., Zhao Z., Shimizu M. (2006). Phenolic Acids Are Absorbed from the Rat Stomach with Different Absorption Rates. J. Agric. Food Chem..

[17] Cianciosi D., Forbes-Hernández T.Y., Giampieri F., Zhang J., Ansary J., Pacetti M., Quiles J.L., Simal-Gandara J., Battino M. (2019). Effect of In vitro Gastrointestinal Digestion on the Bioaccessibility of Phenolic Compounds and Antioxidant Activity of Manuka Honey. eFood.

[18] Peng W., Wang N., Wang S., Wang J., Dong Y. (2022). Effects of exogenous caffeic acid, L-Phenylalanine and NACL treatments on main active components content and in vitro digestion of germinated tartary buckwheat. Foods.

[19] De Paulo Farias D., De Araújo F.F., Neri-Numa I.A., Dias-Audibert F.L., Delafiori J., Catharino R.R., Pastore G.M. (2021). Effect of in vitro digestion on the bioaccessibility and bioactivity of phenolic compounds in fractions of *Eugenia pyriformis* fruit. Food Res. Int..

[20] Tian W., Hu R., Chen G., Zhang Y., Wang W., Li Y. (2021). Potential bioaccessibility of phenolic acids in whole wheat products during in vitro gastrointestinal digestion and probiotic fermentation. Food Chem..

[21] Chateigner-Boutin A., Saulnier L. (2022). Ferulic and coumaric acids in the cereal grain: Occurrence, biosynthesis, biological and technological functions. Advances in Botanical Research.

[22] Ryu D., Koh E. (2016). Influence of cooking methods on free and bound phenolic acids in Korean black rice. J. Food Process. Preserv..

[23] Massaretto I.L., Meza S.L.R., Schmiele M., Marquez U.M.L., Sinnecker P. (2023). Nutritional characterization and effect of cooking on phenolic compounds, antioxidant capacity and sensory acceptability of commercial wild rice (*Zizania aquatica* L.). Biocatal. Agric. Biotechnol..

[24] Călinoiu L.F., Vodnar D.C. (2018). Whole grains and Phenolic acids: A review on bioactivity, functionality, health benefits and bioavailability. Nutrients.

[25] Wang W., Guo J., Zhang J., Peng J., Liu T., Xin Z. (2014). Isolation, identification and antioxidant activity of bound phenolic compounds present in rice bran. Food Chem..

[26] Qiu Y., Liu Q., Beta T. (2009). Antioxidant properties of commercial wild rice and analysis of soluble and insoluble phenolic acids. Food Chem..

[27] Yu X., Yang T., Qi Q., Du Y., Shi J., Liu X., Liu Y., Zhang H., Zhang Z., Yan N. (2021). Comparison of the contents of phenolic compounds including flavonoids and antioxidant activity of rice (*Oryza sativa*) and Chinese wild rice (*Zizania latifolia*). Food Chem..

[28] Chu C., Du Y., Yu X., Shi J., Yuan X., Liu X., Liu Y., Zhang H., Zhang Z., Yan N. (2020). Dynamics of antioxidant activities, metabolites, phenolic acids, flavonoids, and phenolic biosynthetic genes in germinating Chinese wild rice (*Zizania latifolia*). Food Chem..

[29] Colasanto A., Disca V., Travaglia F., Bordiga M., Coïsson J.D., Arlorio M., Locatelli M. (2025). Bioaccessibility of phenolic compounds during simulated gastrointestinal digestion of black rice (*Oryza sativa* L., cv. Artemide). Food Chem..

[30] Wanyo P., Chamsai T., Toontom N., Nghiep L.K., Tudpor K. (2024). Differential effects of in vitro simulated digestion on antioxidant activity and bioaccessibility of phenolic compounds in purple rice bran extracts. Molecules.

[31] Anson N.M., Van Den Berg R., Havenaar R., Bast A., Haenen G.R. (2008). Bioavailability of ferulic acid is determined by its bioaccessibility. J. Cereal Sci..

[32] Aalim H., Arslan M., Abaker H.M.A., Hashim S.B.H., Tahir H.E., Karim N., Shishir M.R.I., Zhai X., Li Z., Zhou C. (2026). Phenolics distribution in rice and their macromolecular interactions: A Matrix-Centric Perspective. Foods.

[33] Pang Y., Ahmed S., Xu Y., Beta T., Zhu Z., Shao Y., Bao J. (2017). Bound phenolic compounds and antioxidant properties of whole grain and bran of white, red and black rice. Food Chem..

[34] White B.L., Howard L.R., Prior R.L. (2010). Release of Bound Procyanidins from Cranberry Pomace by Alkaline Hydrolysis. J. Agric. Food Chem..

[35] Xu Z., Xiong X., Zeng Q., He S., Yuan Y., Wang Y., Wang Y., Yang X., Su D. (2020). Alterations in structural and functional properties of insoluble dietary fibers-bound phenolic complexes derived from lychee pulp by alkaline hydrolysis treatment. LWT.

[36] Hur S.J., Lim B.O., Decker E.A., McClements D.J. (2010). In vitro human digestion models for food applications. Food Chem..

[37] Sumczynski D., Kotásková E., Orsavová J., Valášek P. (2016). Contribution of individual phenolics to antioxidant activity and in vitro digestibility of wild rices (*Zizania aquatica* L.). Food Chem..

[38] Shao Y., Hu Z., Yu Y., Mou R., Zhu Z., Beta T. (2017). Phenolic acids, anthocyanins, proanthocyanidins, antioxidant activity, minerals and their correlations in non-pigmented, red, and black rice. Food Chem..

[39] Yu J., Zheng X., Zhu D., Xu Q., Xu F., Chen M., Meng L., Shao Y. (2024). Changes of polyphenols and their antioxidant activities in non-pigmented, red and black rice during in vitro digestion. Food Chem. X.

[40] Kroon P.A., Faulds C.B., Ryden P., Robertson J.A., Williamson G. (1997). Release of Covalently Bound Ferulic Acid from Fiber in the Human Colon. J. Agric. Food Chem..

[41] Shahidi F., Hossain A. (2023). Importance of Insoluble-Bound phenolics to the antioxidant potential is dictated by source material. Antioxidants.

[42] McGilp L., Castell-Miller C., Haas M., Millas R., Kimball J. (2023). Northern Wild Rice (*Zizania palustris* L.) breeding, genetics, and conservation. Crop Sci..

[43] Brodkorb A., Egger L., Alminger M., Alvito P., Assunção R., Ballance S., Bohn T., Bourlieu-Lacanal C., Boutrou R., Carrière F. (2019). INFOGEST static in vitro simulation of gastrointestinal food digestion. Nat. Protoc..

[44] Wang B., Nie C., Li T., Zhao J., Fan M., Li Y., Qian H., Wang L. (2022). Effect of boiling and roasting on phenolic components and their bioaccessibilities of highland barley. Food Res. Int..

[45] Drawbridge P.C., Apea-Bah F., Hornung P.S., Beta T. (2021). Bioaccessibility of phenolic acids in Canadian hulless barley varieties. Food Chem..

[46] Apea-Bah F.B., Drawbridge P., Beta T. (2022). A Generalized Method for Determining Free Soluble Phenolic Acid Composition and Antioxidant Capacity of Cereals and Legumes. JoVE.

[47] Sánchez-Gutiérrez M., Gómez-García R., Carrasco E., Rodríguez A., Pintado M. (2025). Bioactive potential of Olive Leaf By-Product throughout in vitro gastrointestinal digestion. Foods.

