# Supplementary material for: Influence of Simulated Gastrointestinal Digestion on Phenolic Composition, Bioaccessibility, and Antioxidant Properties of Commercial Wild Rice
Source: Molecules. 2026 Jul 3;31(13):2333. doi: 10.3390/molecules31132333 (PMC13363508; doi:10.3390/molecules31132333)
Supplement: Supplementary file 1 [file molecules-31-02333-s001.zip › molecules-4378013-supplementary.pdf]

Supplementary Information

Influence of Simulated Gastrointestinal Digestion on Phenolic Composition, Bioaccessibility, and Antioxidant Properties of Commercial Wild Rice

Asif Panchbhaya, Daniela D. Herrera-Balandrano, Beverly Too, and Trust Beta*

1 Department of Food and Human Nutritional Sciences, University of Manitoba, Winnipeg, Manitoba, R3T 2N2, Canada.

* Correspondence: trust.bet@umanitoba.ca

Figure S1. Principal component analysis (PCA) score plot with 95% confidence ellipses for wild rice samples by processing and digestion phase.

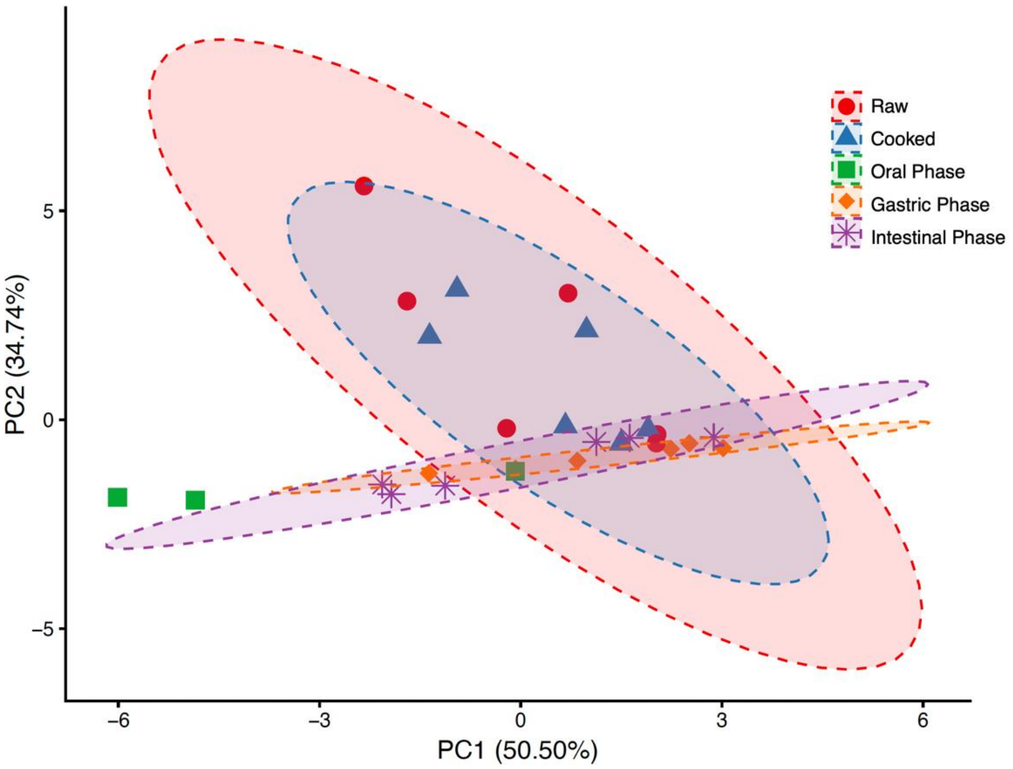

Table S1. PCA Sample Scores

Sample	PC1	PC2	Label
**EG_Raw_Free**	-2.33636161	5.593642999	Raw
**EG_Raw_Bound**	-0.209952729	-0.203869497	Raw
**EG_Cooked_Free**	-0.949411476	3.121395002	Cooked
**EG_Cooked_Bound**	0.666800457	-0.148639292	Cooked
**EG_Oral_Bound**	-4.850769266	-1.926659059	Oral
**EG_Gastric_Free**	2.237037309	-0.695945274	Gastric
**EG_Gastric_Bound**	-0.076574816	-1.174562315	Gastric
**EG_Intestinal_Free**	1.126837682	-0.529624743	Intestinal
**EG_Intestinal_Bound**	-1.928662878	-1.779663255	Intestinal
**SY_Raw_Free**	0.706531017	3.029554733	Raw
**SY_Raw_Bound**	2.034591705	-0.351006691	Raw
**SY_Cooked_Free**	0.981330163	2.147258571	Cooked
**SY_Cooked_Bound**	1.901155945	-0.226561476	Cooked
**SY_Oral_Bound**	-0.079834012	-1.230833364	Oral
**SY_Gastric_Free**	3.019684127	-0.678158373	Gastric
**SY_Gastric_Bound**	0.844338122	-0.982172156	Gastric
**SY_Intestinal_Free**	2.878120826	-0.417201694	Intestinal
**SY_Intestinal_Bound**	-1.125367907	-1.575367294	Intestinal
**FL_Raw_Free**	-1.695524666	2.839110597	Raw
**FL_Raw_Bound**	2.021644295	-0.560242644	Raw
**FL_Cooked_Free**	-1.358450783	1.993719006	Cooked
**FL_Cooked_Bound**	1.497252942	-0.56537307	Cooked
**FL_Oral_Bound**	-6.012321904	-1.855647911	Oral
**FL_Gastric_Free**	2.515211365	-0.562563239	Gastric
**FL_Gastric_Bound**	-1.369750647	-1.280248005	Gastric
**FL_Intestinal_Free**	1.626687466	-0.434559408	Intestinal
**FL_Intestinal_Bound**	-2.064240726	-1.54578215	Intestinal

Principal component scores (PC1 and PC2) for each wild rice (WR) sample, with sample labels indicating variety, state, and fraction.

Table S2. PCA Variable Loadings

Variable	PC1	PC2
**DPPH**	-0.112477325	0.939179288
**ABTS**	-0.169245592	0.962970337
**TPC**	-0.727922954	0.618699361
**Ferulic acid**	-0.5759522	-0.51915108
**Gallic acid**	-0.312349292	0.877086977
**Sinapic acid**	-0.842552484	-0.376477213
**Vanillic acid**	-0.927210821	-0.242666632
**p-coumaric**	-0.950186314	-0.001078487
**p-Hydroxybenzoic acid**	-0.894411202	0.032006212
**Caffeic acid**	-0.881298813	-0.203482208

Loadings represent the correlation coefficients between each measured variable and the principal components PC1 and PC2.

Table S3. Contribution of Variables to Principal Components

Contribution of Variables	PC1	PC2
**DPPH**	0.002505387	0.25390185
**ABTS**	0.005672568	0.266928331
**TPC**	0.10493389	0.110186449
**Ferulic acid**	0.065692808	0.077581179
**Gallic acid**	0.01932084	0.221439033
**Sinapic acid**	0.140584962	0.040798692
**Vanillic acid**	0.170255804	0.016950752
**p-coumaric**	0.178797929	3.3481E-07
**p-Hydroxybenzoic acid**	0.158423426	0.000294875
**Caffeic acid**	0.153812387	0.011918504

Contribution values indicate the proportion of each variable's influence on the variance explained by PC1 and PC2.

Table S4. PERMANOVA results for statistical validation of group separation in PCA space.

Source	Df	Sum Sq	R^2	F	P (perm)
**State/Digestion phase**	4	94.096	0.425	4.06	0.003**
**Residual**	22	127.518	0.575		
**Total**	26	221.613	1.000		

*Df = degrees of freedom; Sum Sq = sum of squares; R^2 = proportion of total variance explained; F = pseudo-F by permutation; P (perm) = permutation p-value (999 permutations). PERMANOVA results based on testing for group separation by processing/digestion phase (Label: Raw, Cooked, Oral, Gastric, Intestinal). Significance codes: ***$p < 0.001$; **$p < 0.01$; *$p < 0.05$.

Table S5. Normality Tests for Phenolic compounds, TPC, and Antioxidant Activities

Shapiro-Wilk test	DPPH	ABTS	TPC	Ferulic acid	Gallic acid	Sinapic acid	Vanillic acid	p-coumaric	p-Hydroxybenzoic acid	Caffeic acid
W	0.89	0.88	0.98	0.93	0.94	0.83	0.83	0.9	0.92	0.86
P value	0.149	0.116	0.9842	0.4037	0.5058	0.0332	0.0313	0.197	0.3961	0.0872
Passed normality test (alpha=0.05)?	Yes	Yes	Yes	Yes	Yes	No	No	Yes	Yes	Yes
P value summary	ns	ns	ns	ns	ns	*	*	ns	ns	ns

Anderson-Darling test	DPPH	ABTS	TPC	Ferulic acid	Gallic acid	Sinapic acid	Vanillic acid	p-coumaric	p-Hydroxybenzoic acid	Caffeic acid
A2	0.51	0.55	0.18	0.34	0.31	0.76	0.82	0.47	0.36	0.61
P value	0.1483	0.1176	0.8828	0.4262	0.4955	0.0307	0.0217	0.1895	0.373	0.0829
Passed normality test (alpha=0.05)?	Yes	Yes	Yes	Yes	Yes	No	No	Yes	Yes	Yes
P value summary	ns	ns	ns	ns	ns	*	*	ns	ns	ns

ns, not significant ($p \geq 0.05$); *, $p < 0.05$. W, Shapiro–Wilk test statistic; A2, Anderson–Darling test statistic. Normality was assessed using the Shapiro–Wilk and Anderson-Darling tests ($\alpha = 0.05$). Variables with $p \geq 0.05$ are considered to have passed the normality test, indicating no significant deviation from normality; $p < 0.05$ indicates failure of the normality test.

Table S6. Pearson Correlation Coefficients Among Phenolic Compounds, TPC, and Antioxidant Activities

Pair	r	Raw p-value	FDR-adjusted p-value	Significance
**DPPH vs ABTS**	0.98	2.49×10^{-19}	1.12×10^{-17}	***
**DPPH vs TPC**	0.64	2.90×10^{-4}	0.0087	*
**DPPH vs Ferulic acid**	-0.37	0.0592	0.11	
**DPPH vs Gallic acid**	0.75	6.41×10^{-6}	0.00029	***
**DPPH vs Sinapic acid**	-0.21	0.2979	0.38	
**DPPH vs Vanillic acid**	-0.13	0.518	0.65	
**DPPH vs p-coumaric**	0.12	0.5562	0.63	
**DPPH vs p-Hydroxybenzoic acid**	0.07	0.7256	0.81	
**DPPH vs Caffeic acid**	-0.09	0.6439	0.79	
**ABTS vs TPC**	0.72	2.32×10^{-5}	0.00074	***
**ABTS vs Ferulic acid**	-0.33	0.0943	0.17	
**ABTS vs Gallic acid**	0.84	5.08×10^{-8}	0.0000023	***
**ABTS vs Sinapic acid**	-0.21	0.3031	0.37	
**ABTS vs Vanillic acid**	-0.06	0.7571	0.82	
**ABTS vs p-coumaric**	0.16	0.4178	0.51	
**ABTS vs p-Hydroxybenzoic acid**	0.13	0.5192	0.62	
**ABTS vs Caffeic acid**	-0.07	0.734	0.81	
**TPC vs Ferulic acid**	0.19	0.3392	0.42	
**TPC vs Gallic acid**	0.79	1.16×10^{-6}	0.000036	***
**TPC vs Sinapic acid**	0.38	0.0519	0.1	
**TPC vs Vanillic acid**	0.55	0.00326	0.029	*
**TPC vs p-coumaric**	0.64	0.00037	0.0091	*
**TPC vs p-Hydroxybenzoic acid**	0.59	0.00108	0.022	*
**TPC vs Caffeic acid**	0.50	0.00826	0.049	*
**Ferulic acid vs Gallic acid**	-0.28	0.1509	0.24	
**Ferulic acid vs Sinapic acid**	0.61	0.0008	0.022	*
**Ferulic acid vs Vanillic acid**	0.76	4.31×10^{-6}	0.00014	***
**Ferulic acid vs p-coumaric**	0.52	0.00511	0.039	*
**Ferulic acid vs p-Hydroxybenzoic acid**	0.32	0.1084	0.17	
**Ferulic acid vs Caffeic acid**	0.44	0.0232	0.055	
**Gallic acid vs Sinapic acid**	-0.13	0.5024	0.65	
**Gallic acid vs Vanillic acid**	0.10	0.624	0.73	
**Gallic acid vs p-coumaric**	0.29	0.1453	0.23	
**Gallic acid vs p-Hydroxybenzoic acid**	0.36	0.0681	0.13	
**Gallic acid vs Caffeic acid**	0.07	0.737	0.81	
**Sinapic acid vs Vanillic acid**	0.80	6.87×10^{-7}	0.000023	***
**Sinapic acid vs p-coumaric**	0.80	5.88×10^{-7}	0.000022	***

**Sinapic acid vs p-Hydroxybenzoic acid**	0.74	1.00×10^{-5}	0.00028	***
**Sinapic acid vs Caffeic acid**	0.83	1.08×10^{7}	0.0000049	***
**Vanillic acid vs p-coumaric**	0.86	9.92×10^{-9}	0.00000044	***
**Vanillic acid vs p-Hydroxybenzoic acid**	0.76	3.36×10^{6}	0.00012	***
**Vanillic acid vs Caffeic acid**	0.84	3.23×10^{-8}	0.0000016	***
**p-coumaric vs p-Hydroxybenzoic acid**	0.87	3.79×10^{-9}	0.00000017	***
**p-coumaric vs Caffeic acid**	0.80	5.15×10^{-7}	0.000018	***
**p-Hydroxybenzoic acid vs Caffeic acid**	0.80	5.07×10^{-7}	0.000019	***

Pearson correlation coefficients (r), raw p-values, and Benjamini–Hochberg FDR-adjusted p-values are shown for each pairwise comparison. Significance is indicated as follows: $p < 0.05$ (*), $p < 0.01$ (**), $p < 0.001$ (***), based on FDR-adjusted p-values.
